# Supplementary figures and images for: Structural basis of HapEP88L-linked antifungal triazole resistance in Aspergillus fumigatus
Source: Life Sci Alliance. 2020 May 28;3(7):e202000729. doi: 10.26508/lsa.202000729 (PMC7266990; doi:10.26508/lsa.202000729)

Source Data for Figure 1

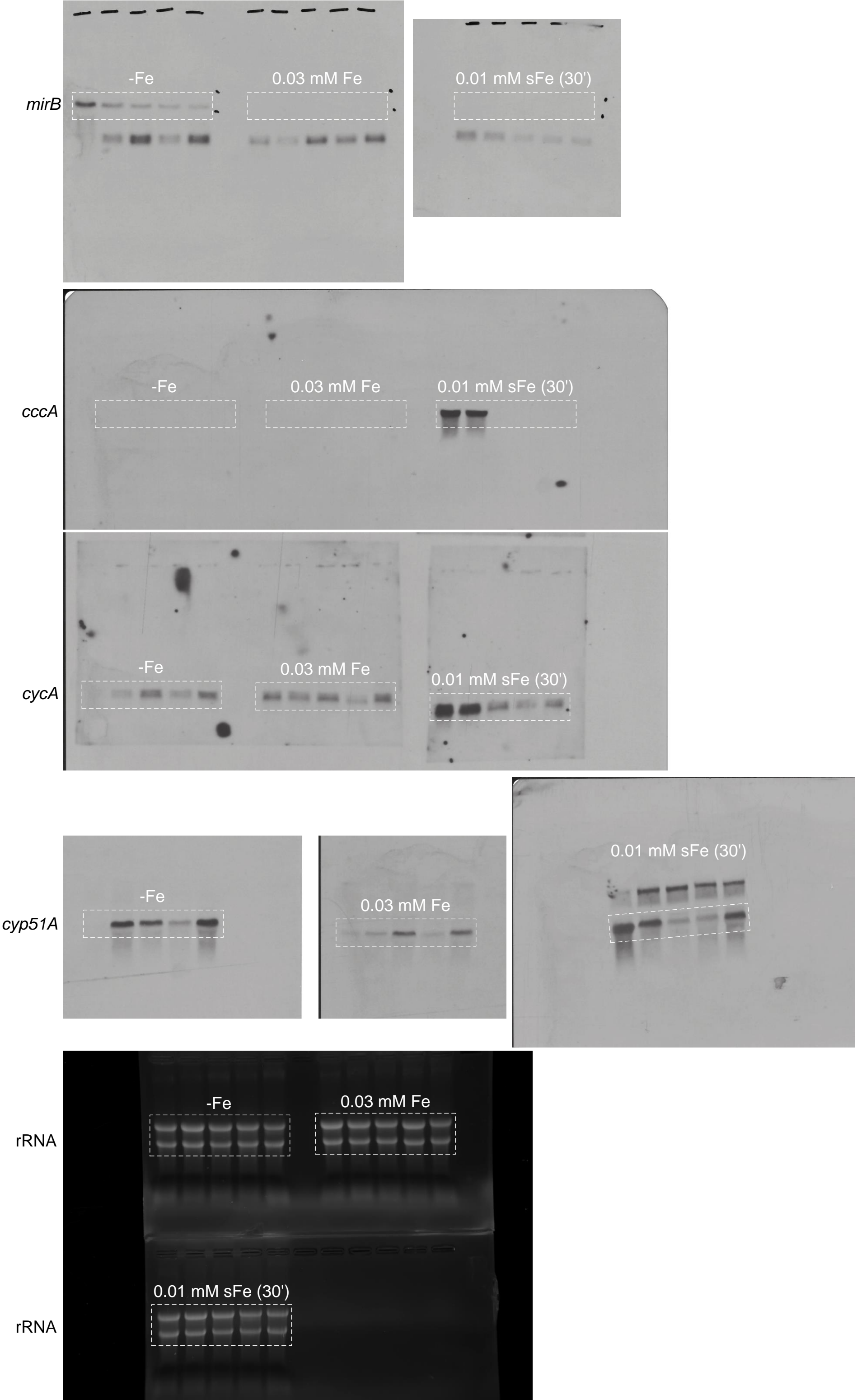

Supplement: Supplementary file 1 [file LSA-2020-00729_SdataF1.pdf]

Source Data for Figure 4

Figure 4A

Figure 4B

Figure 4C

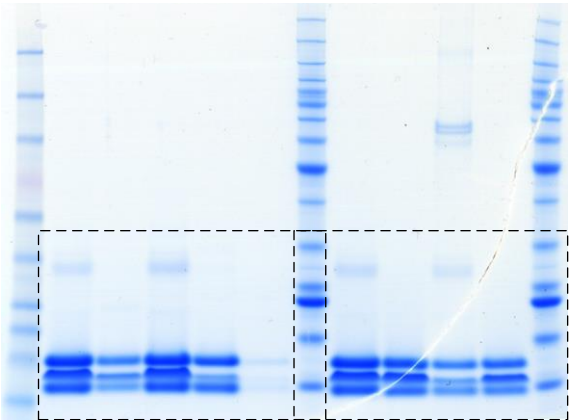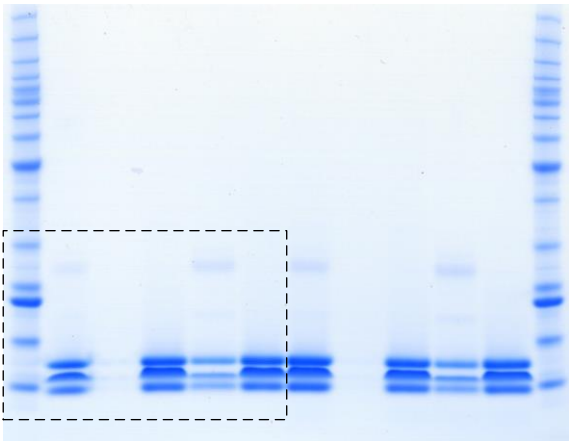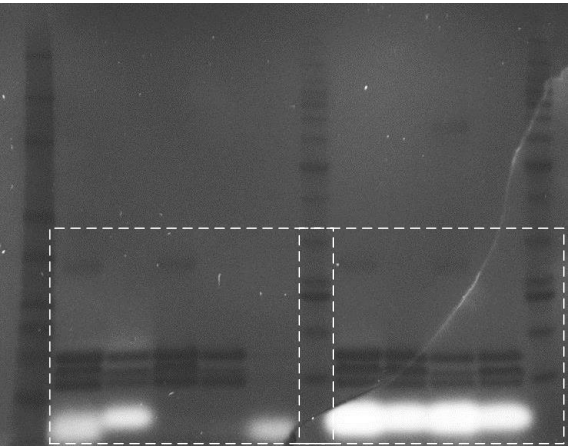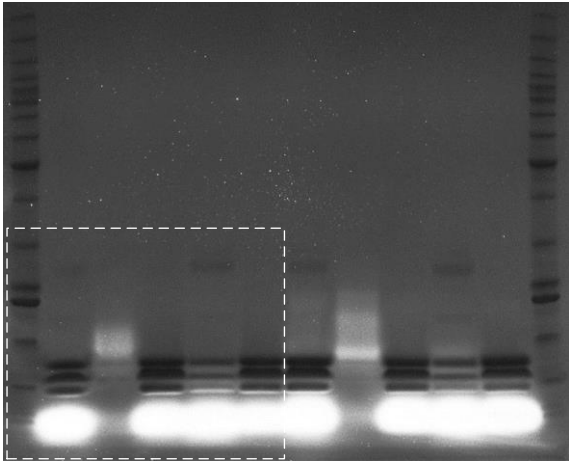

Supplement: Supplementary file 2 [file LSA-2020-00729_SdataF4.pdf]
